# Interaction of Carbohydrate Coated Cerium-Oxide Nanoparticles with Wheat and Pea: Stress Induction Potential and Effect on Development

**DOI:** 10.3390/plants8110478

**Published:** 2019-11-06

**Authors:** Ivana Milenković, Aleksandra Mitrović, Manuel Algarra, Juan M. Lázaro-Martínez, Enrique Rodríguez-Castellón, Vuk Maksimović, Slađana Z. Spasić, Vladimir P. Beškoski, Ksenija Radotić

**Affiliations:** 1 Institute for Multidisciplinary Research, University of Belgrade, KnezaVišeslava 1, 11030 Belgrade, Serbia; ivana.milenkovic@imsi.rs (I.M.); mita@imsi.rs (A.M.); maxivuk@imsi.rs (V.M.); sladjana@imsi.rs (S.Z.S.); 2 CQM-Centro de Química da Madeira, University of Madeira, Campus da Penteada, 9020-105 Funchal, Portugal; 3 Facultad de Farmacia y Bioquímica, UNIVERSIDAD de Buenos Aires, IQUIMEFA-CONICET, Junín 956 (C1113AAD), CABA, Argentina; jmlazaromartinez@gmail.com; 4 Department of Inorganic Chemistry, University of Málaga, 29007 Málaga, Spain; 5 Singidunum University, Danijelova 32, 11010 Belgrade, Serbia; 6 Faculty of Chemistry, University of Belgrade, Studentskitrg 12-16, 11000 Belgrade, Serbia; vbeskoski@chem.bg.ac.rs

**Keywords:** germination, growth, nanomaterial, characterization, plant, phenolic profile, total phenolic content, total antioxidative activity

## Abstract

Reports about the influence of cerium-oxide nanoparticles (nCeO_2_) on plants are contradictory due to their positive and negative effects on plants. Surface modification may affect the interaction of nCeO_2_ with the environment, and hence its availability to plants. In this study, the uncoated and glucose-, levan-, and pullulan-coated nCeO_2_ were synthesized and characterized. The aim was to determine whether nontoxic carbohydrates alter the effect of nCeO_2_ on the seed germination, plant growth, and metabolism of wheat and pea. We applied 200 mgL^−1^ of nCeO_2_ on plants during germination (Ger treatment) or three week-growth (Gro treatment) in hydroponics. The plant response to nCeO_2_ was studied by measuring changes in Ce concentration, total antioxidative activity (TAA), total phenolic content (TPC), and phenolic profile. Our results generally revealed higher Ce concentration in plants after the treatment with coated nanoparticles compared to uncoated ones. Considering all obtained results, Ger treatment had a stronger impact on the later stages of plant development than Gro treatment. The Ger treatment had a stronger impact on TPC and plant elongation, whereas Gro treatment affected more TAA and phenolic profile. Among nanoparticles, levan-coated nCeO_2_ had the strongest and positive impact on tested plants. Wheat showed higher sensitivity to all treatments.

## 1. Introduction

The production of cerium-oxide nanoparticles (nCeO_2_) totals around 10,000 metric tons per year, making them one of the most produced metal oxide nanoparticles [1]. Cerium-oxide nanoparticles have become a popular nanomaterial due to their unique redox properties based on their facile transition between Ce^3+^ and Ce^4+^ oxidation states [2]. Their use is increasing in the pharmaceutical industry, electronics, cosmetics, paints, fuel additives, and petrochemical processing [3,4,5,6,7]. Thus, the nanoparticles can be found in the environment, and analysis of their ecotoxicity is needed. To improve their solubility, the coating of nCeO_2_ with different polymers has been performed by many researchers [8,9]. The studies reported on positive and negative effects on different plants of uncoated nCeO_2_ and nCeO_2_ coated with various organic compounds are presented in Supplementary Table S1. As can be seen, data on the effect of coated nCeO_2_ on hydroponically cultivated plants are scarce.

Since polysaccharides are natural compounds and carbohydrate-coated nCeO_2_ have not been used for extended studies on plants, it is worth analyzing their effect and comparing it with the effect of uncoated nCeO_2_. In this study, nCeO_2_ were coated with glucose as a monosaccharide and previously used coating agent [10], as well as with microbiologically produced exopolysaccharides, levan ((2→6)-β-D-fructan) and pullulan (α(1→6)-glucan). Their effect on the development and stress of two chosen plant species was studied. Levan and pullulan were selected as nontoxic polysaccharides and are increasingly applied in biomedicine [11,12] and the food industry [13,14] with the ability of film formation [15,16]. The key point of the mechanism of nCeO_2_ coating with glucose is the reduction of Ce^4+^ to Ce^3+^ [10], while polysaccharides have a greater tendency to complex Ce^4+^ due to the higher number of hydroxyl groups. Based on the available literature, the microbial exopolysaccharide pullulan was used for the first time as a nanoparticle coating agent. The effect of 200 mgL^−1^ uncoated (nCeO_2_) and glucose-, levan-, and pullulan-coated nCeO_2_ (G-CeO_2_, L-CeO_2_, P-CeO_2_) was studied on two plant species–one monocotyledonous, wheat (*Triticum aestivum* L.), and one dicotyledonous, pea (*Pisum sativum* L.)–in order to determine whether there is a difference in their response to nanoparticle treatment.

The first goal was to find out if coating with natural carbohydrates affects Ce concentration in plants by testing the effect of uncoated and coated nCeO_2_ applied during germination (Ger treatment) and during growth (Gro treatment) in hydroponic culture. In relation to this, we examined whether coated nCeO_2_ had a toxic or stimulating effect on plants, keeping in mind that CeO_2_ is an integral part of some fertilizers’ composition [17]. Hydroponics was chosen because it allows higher nanoparticles’ uptake compared to soil, where various interactions between an organic compound in the coating and organic compounds in the soil affect the uptake [18]. The second goal was to see whether Ger treatment exhibits effects on germination or on the later stages of plant development, which may have application in nano-priming technology, to enhance seedling growth and stress resistance. The total phenolic content (TPC), phenolic profile and total antioxidative activity (TAA) were measured as indicators of plant response to nanoparticles.

## 2. Results and Discussion

### 2.1. Cerium-Oxide Nanoparticles Structural Properties

**Figure 1:**
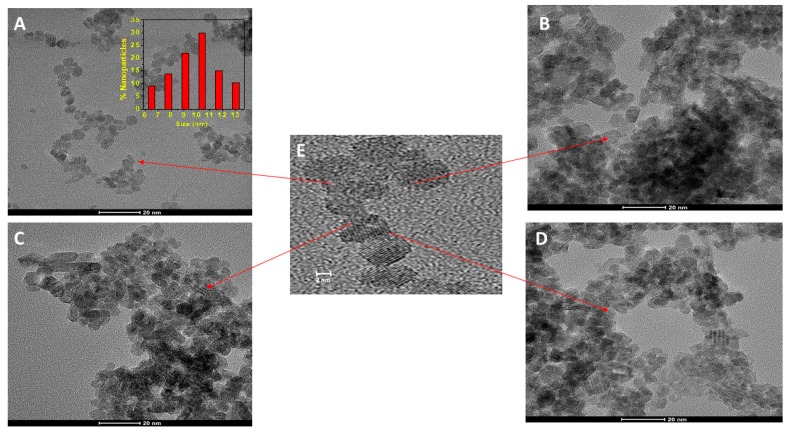
Transmission electron microscopy (TEM) images of the obtained nCeO_2_: (**A**) Uncoated cerium-oxide nanoparticles (nCeO_2_), (**B**) G-CeO_2_, (**C**) L-CeO_2_, and (**D**) P-CeO_2_. (**E**) Magnified high-resolution transmission electron microscopy (HRTEM) image of the lattice space in the nCeO_2_. Histogram of nanoparticles size distribution is shown as the inset in (**A**).

The physicochemical characteristics of the synthesized nCeO_2_ were analyzed by high-resolution transmission electron microscopy (HRTEM), X-ray diffraction (XRD), and X-ray photoelectron spectroscopy (XPS), as well as hydrogen nuclear magnetic resonance (^1^H) and carbon-13 nuclear magnetic resonance (^13^C-NMR). Uncoated nCeO_2_ (Figure 1A) and coated, G-, L-, and P-CeO_2_, were analyzed by HRTEM (Figure 1B–D). The results of Z-average particle size, the intensity distribution of particle size, zeta potential, Fourier-transform infrared (FTIR) spectra, and turbidity for these nanoparticles were previously analyzed [9].

The HRTEM images for uncoated nCeO_2_ demonstrated an average size between 4–5 nm It can be seen from the magnified HRTEM image in the center (Figure 1E) that the nanoparticles’ diameter after coating with the different carbohydrate increased twice, with sizes between 8–13 nm. Also, the HRTEM images showed a crystalline structure with a spacing of 0.333 nm (Figure 1E) in all of the used nCeO_2,_ which indicates that all obtained nCeO_2_ had a face-centered cubic crystallographic structure [19].

**Figure 2:**
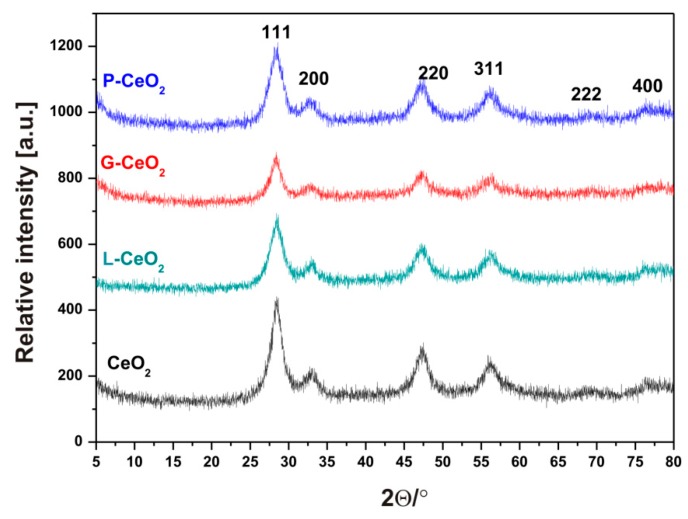
X-ray diffraction (XRD) patterns of the uncoated and three different coated nCeO_2_.

The obtained XRD patterns of nCeO_2_ are shown in Figure 2. The samples showed typical peaks corresponding to 111, 200, 220, 311 planes, which are typical of face-centered structures. Broad peaks were observed for all samples, which was due to their synthesis at low temperature. However, the shifts were not found even after the further coating process, meaning that crystalline structure remained unchanged.

The XPS measurements further verified the successful formation of nCeO_2_ and surface coated nCeO_2_. The determined elemental compositions of the nCeO_2_ surface and anchor layers are reported in Table 1, which summarizes the elemental composition (atomic %, at. %) calculated from the Ce 3*d*, C 1*s*, and O 1*s* collected for uncoated and coated nCeO_2_.

**Figure 3:**
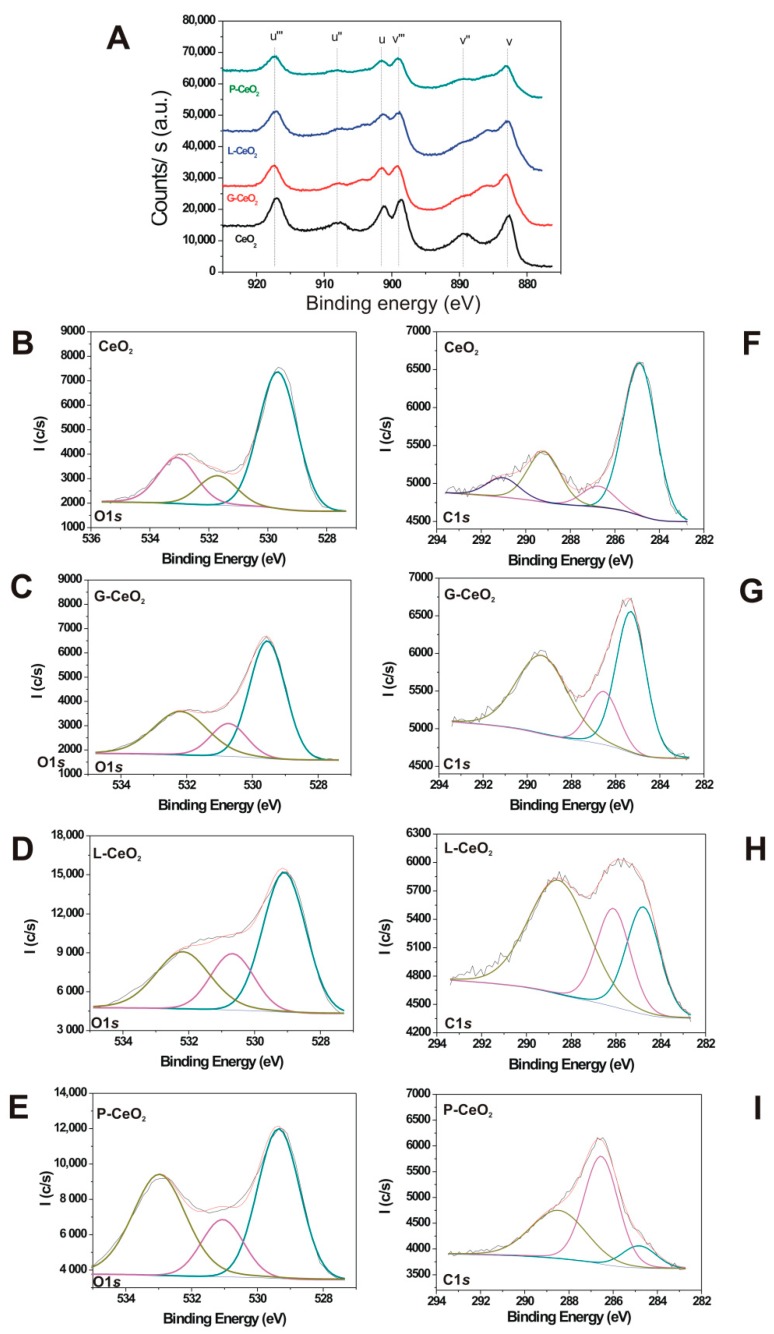
High-resolution X-ray photoelectron spectroscopy (XPS) spectra for the uncoated nCeO_2_ and coated nanoparticles: Ce 3*d* (**A**), O 1*s* for CeO_2_ (**B**), G-CeO_2_ (**C**), L-CeO_2_ (**D**) and P-CeO_2_ (**E**) and C 1*s* core level spectra for CeO_2_ (**F**), G-CeO_2_ (**G**), L-CeO_2_ (**H**) and P-CeO_2_ (**I**). The different colors show the assignment of different core levels.

The hydrogen bonding between oxygen atoms of CeO_2_ structure and the organic coating caused an increase in the contributions of at. % C and was associated with a significant decrease in the surface concentration of CeO_2_. The Ce 3*d* core level spectra of coated samples also showed clear modifications due to the formation of Ce^3+^ (Figure 3A). The new contributions at 880.6 eV, 884.8 eV, 899.0 eV, and 902.5 eV were found, which are characteristic of Ce^3+^ [20]. As expected, the interaction of the studied carbohydrates with nCeO_2_ produced a partial reduction of Ce^4+^, while the samples L-CeO_2_ and G-CeO_2_ were those with a higher reduction degree of Ce^4+^.

Figure 3B shows the O 1*s* core level. The main peak at 529–530 eV was associated with lattice oxygen of ceria and assigned to the metal oxide (Ce-O) binding energy, and the second one at 531.5–532 eV was due to the oxygen vacancies [21]. The signal due to oxygen from carbonate was overlapped with the main signal at 529–530 eV. The O 1*s* core level spectra of the coated samples (Figure 3C–E) showed some changes in comparison to that of uncoated nCeO_2_. The relative intensity of their contributions, at about 532.0 eV, was higher due to the oxygen from C-OH, and C-O-C of L-CeO_2_ and P-CeO_2_.

Figure 3F shows the C 1*s* core level for the uncoated nCeO_2_, which can be resolved into different contributions. The main contribution was centered at 284.8 eV, and can be assigned to sp^3^ (adventitious carbon) (C-C and C-H) and sp^2^ (-C=C-). The C-O contribution of hydroxyl groups (at 286.5 eV) was found in the coated nCeO_2_. As expected, nCeO_2_ showed contributions at 289.3 eV due to the formation of surface cerium carbonate. The fourth contribution at 291.2 eV was assigned to the satellite π→π* due to double bond delocalization [22].

The C 1*s* core level spectra for coated samples (Figure 3G–I) showed clear changes due to the coating, with high-intensity contributions due to ethanolic (C-OH) and etheric (C-O-C) groups at about 286.3 eV, and carboxylate and carbonate groups at about 289.0 eV. Finally, the broad signal at higher binding energy may be mainly assigned to the Na KLL signal, which overlapped with the contribution of oxygen from water.

The materials that contain paramagnetic ions are not generally studied through solid-state nuclear magnetic resonance (ssNMR) since the resonance signals related to the closest atoms to these ions are extremely affected. The common nuclei studied in the organic matrix that carry these paramagnetic species cannot be detected, or the resonance signals are highly affected since the relaxation process is enhanced. For this reason, the direct polarization or cross-polarization strategies commonly employed in the acquisition of ^13^C ss-NMR experiments for structural purposes can detect which segments/regions of the polymeric material are close to the paramagnetic entities. With this aim, different authors have been used NMR as an approach to find out which ligands present in polymeric networks are involved in the coordination of Cu [23,24], Hg [25], Co [23,26], and Sm [27] ions in non-crystalline systems.

### 2.2. Cerium Concentration and Translocation in Plants

Considering morphological parameters, the studied plant species showed no visible signs of damage induced by treatments, such as necrosis, chlorosis, or stunting. The significant difference in the Ce concentration in plants between Ger and Gro treatments, shown in Table 2, was expected, taking into account the difference in treatment duration (Ger treatment: 3–5 days; Gro treatment: 21 days). Also, the Ce concentration in plants after the treatments was different in the two plant species. In both Ger and Gro treatments, a higher Ce concentration was found in wheat. The high Ce concentration in wheat shoots after Gro treatment differs from the literary results [28] (Supplementary Table S1) and could be attributed to the longer exposure time to nCeO_2_ in our experiments. Although G-CeO_2_ is one of the most stable nCeO_2_ [9], their concentration in plants was the lowest after Ger treatments. This indicates that nanoparticles’ suspension stability does not necessarily affect Ce concentration in plants. After the treatments with carbohydrate-coated Ce nanoparticles, the Ce concentration in plants was significantly different compared to uncoated ones (Table 2). After treatments with coated nCeO_2_, the higher Ce concentration in the wheat and pea roots may be due to the higher suspension stability of coated nanoparticles. The coating of nCeO_2_ led to a decrease of Ce concentration in the wheat shoot in both Ger and Gro treatments, as reported in the literature [28] (Supplementary Table S1). It is important to notice that Ce concentration in a plant depends on the interaction degree of the root system with the exposure solution, and is therefore related to growth kinetics, root architecture, and nano-sedimentation [18].

In Gro treatments, the Ce accumulation was considerably higher in wheat than in pea (Table 2). However, the Ce translocation from the roots to shoots was higher in pea than in wheat in most treatments, regardless of nanoparticle coating (Supplementary Figure S2). In Gro treatments, the P-CeO_2_ reached 20 times higher in pea compared to wheat. Compared to uncoated nCeO_2_, the increased Ce concentration for coated nCeO_2_ in pea shoots and decreased Ce concentration in wheat shoots can be explained by higher Ce translocation in pea compared to wheat. Since there is no interaction of nanoparticles with soil in hydroponic culture, the difference in Ce concentration and translocation in wheat and pea could be attributed to the differences in their root anatomy (such as the structure of apoplastic barriers and root branching) and mechanisms of metal distribution [29]. In Ger treatment, the translocation of Ce occurred only in wheat (Supplementary Figure S2).

### 2.3. Effect of nCeO_2_ on Seed Germination and Plant Growth

The literary data reveal either positive or negative effects of nCeO_2_ on plants (Supplementary Table S1), which might depend on the plant species, cultivation method, concentration of nanoparticles, and coating material. Various factors, such as physicochemical properties of nanoparticles, stage of plant development, and properties of culture media influence the phytotoxicity of nCeO_2_. The cultivation method can have an important effect on Ce concentration in plants and therefore phytotoxicity, such as the reported increased uptake of nCeO_2_ using the hydroponic system for plant growth [30].

In order to compare the susceptibility of plants in different stages of development to nCeO_2_, we studied three-week-old plants grown in hydroponics treated with nanoparticles during two different developmental phases: during seed germination (Ger treatment) and seedling growth in hydroponics (Gro treatment). According to the literary data (Supplementary Table S1), nCeO_2_ can show the opposite effects on root growth when applied in low (stimulated) [31] or high concentrations (inhibited) [32]. In this work, a concentration of 200 mgL^−1^ was chosen as a concentration which is in the lower range of literary reported concentrations for hydroponics (10–10,000 mgL^−1^) [18].

Our results showed that none of the nCeO_2_ types in the tested concentration (200 mgL^−1^) significantly affected seed germination in either of the two plant species (*p* < 0.05, Supplementary Figure S3). The germination rate, in the presence of uncoated or coated nCeO_2_, did not vary in comparison with the control for both plant species.

**Figure 4:**
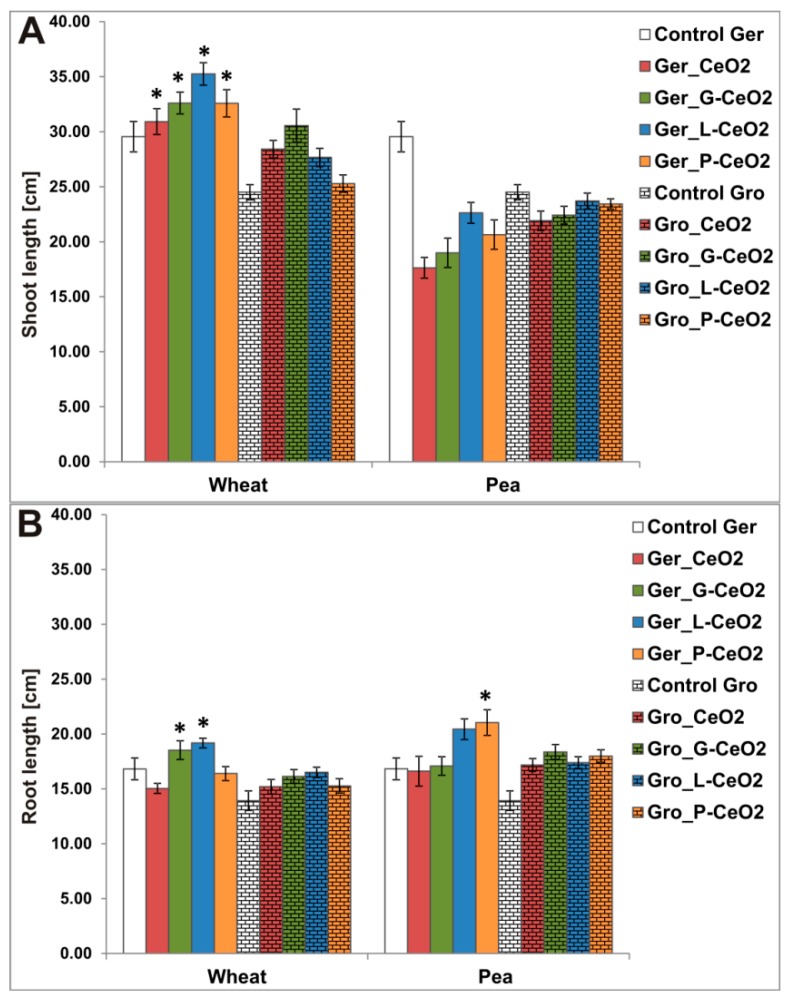
Shoot (**A**) and root elongation (**B**) in wheat and pea after Ger and Gro treatment with different nCeO_2_. Values are shown as mean ± SE; * indicates a statistically significant difference compared to the corresponding control, *p* < 0.05.

Plant growth was not affected by nanoparticles in Gro treatment, but Ger treatment affected shoot and root growth in further phases of seedling development (Figure 4A,B). However, there was no effect on seed germination rate.

Regarding Ger treatments with all nCeO_2_ types, shoot elongation was significantly stimulated only in wheat (Figure 4A). Root elongation (Figure 4B) was also significantly stimulated by Ger treatments Ger_G-CeO_2_ and Ger_L-CeO_2_ in wheat and by Ger_P-CeO_2_ in pea.

Contrary to the Ger treatment, the Gro treatment did not affect plant elongation despite longer exposure to nCeO_2_ (four days in Ger treatment vs. three weeks in Gro treatment) and thus higher Ce accumulation in plants. This indicates that germination is a more sensitive phase of development to nCeO_2_ than growth and that tested nCeO_2_ were nontoxic. Both tested plant species were susceptible to nCeO_2_ treatments during seed germination, the earliest stage of development. Seed germination percentage/rate was unaffected, while shoot and root growth were affected in further phases of seedling development. The absence of the effect of nCeO_2_ treatments on seed germination could be explained as follows: in plant ontogenesis, germination is the first phase, and thus, its regulation is enabled by the information from the seed at the period when the seedling is still unable to experience the environment without comparison with the mother plant’s information [33]. Our results indicated that the wheat shoot was more affected by Ger treatment than the pea.

### 2.4. Antioxidative Response to nCeO_2_ Treatments

Monitoring of secondary metabolites is an essential parameter in the examination of plant response to metal stress. Secondary metabolites are produced under biotic and abiotic stress and help plants to overcome this state and to adapt to the environment [34,35]. Under stress, plants produce free radicals, which damage biomolecules like lipids, DNA, and proteins [34,36]. To alleviate the harmful effect of free radicals, plants developed efficient enzymatic and non-enzymatic defense systems—low molecular weight antioxidants—such as vitamins, phenolic acids, etc. [34,36]. TAA comprises the contribution of different non-enzymatic components with antioxidant capacity (ascorbate, glutathione, phenolics, sugars, etc.) and may be an indicator of the metabolic disorder in plants. Phenolics, as a group of secondary metabolites, are one of the most diverse plant active substances which participate in the regulation of seed germination, plant growth, and in defense responses [34,37]. Due to their redox properties, phenolic compounds have high antioxidant capacity, enabling them to act as hydrogen donors, singlet oxygen quenchers, reducing agents, or metal chelators [38]. Phenolic compounds also act as signaling molecules [39]. Therefore, in this study, we compared TPC, phenolic profile, and TAA as a part of non-enzymatic defense systems and indicators of plants’ response to nanoparticles in both roots and shoots after treatment with various nCeO_2_.

**Figure 5:**
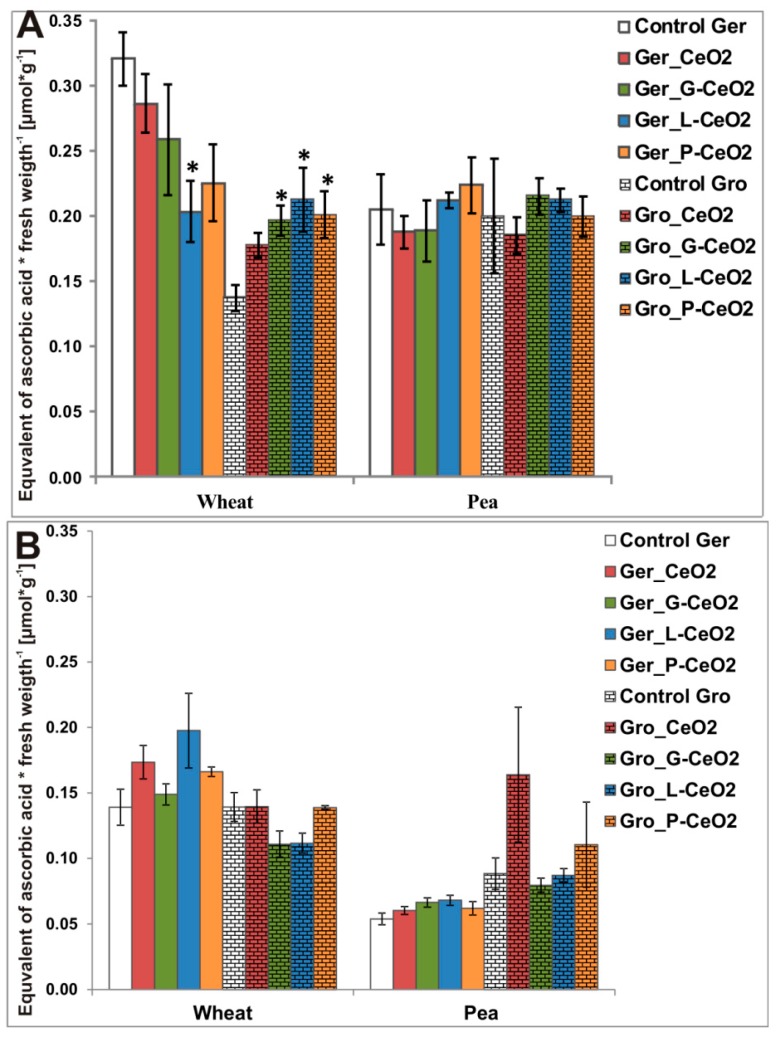
Effect of different nCeO_2_ on total antioxidative activity (TAA) in wheat and pea shoots (**A**) and roots (**B**) after Ger and Gro treatment. Values are shown as mean ± SE; * indicates statistically significant differences in comparison with the corresponding control, *p* < 0.05.

TAA content was measured in the shoots and roots of the nCeO_2_-treated plants. Only wheat showed changes in TAA (Figure 5). The results indicated that nCeO_2_ had an opposite effect on wheat applied during Ger treatment compared to Gro treatment. Compared to the control (0.32 µmolg^−1^ for Ger and 0.14 µmolg^−1^ for Gro treatment), TAA significantly increased only after Gro treatments with coated nCeO_2_ (0.20, 0.21 and 0.20 µmolg^−1^ for G-, L- and P-CeO_2_, respectively) and decreased after Ger_L-CeO_2_ (0.20 µmolg^−1^) in the wheat shoot (Figure 5). This may be in accordance with higher Ce concentration observed in wheat (Table 2). The TAA in roots was not significantly affected after both Ger and Gro treatments.

The nCeO_2_ can have dual activity—reactive oxygen species (ROS) producing [40] or ROS scavenging [41]—due to the presence of 4*f* electrons in Ce and the possibility of reverse transformation between Ce^3+^ and Ce^4+^. The TAA reduction after Ger treatment in the wheat shoot (Figure 5) may indicate ROS scavenging activity of nCeO_2_. Contrary to that, nCeO_2_ may induce excessive production of ROS [42], which may activate the plant defense system, leading to an increase of TAA in the wheat shoot after Gro treatment (Figure 5A). The opposite effect of the Ger treatment compared to the Gro treatment might be attributed to the different behavior of Ce in different plant species, which depends on the surrounding conditions in particular plant species, such as pH and the presence of different kinds of small molecules, etc., as well as the characteristics of the coating agents [43]. Among the nanoparticles, L-CeO_2_ affected TAA the most, confirming that coating can modify nanoparticles’ effect on plants. The coating of nCeO_2_ had a higher impact on antioxidant activity in wheat after Gro treatment (all coated nCeO_2_ affected TAA) compared with the Ger treatment (only L-CeO_2_ affected TAA). The expressed effect of coated nanoparticles may be related to their higher Ce^4+^ reduction degree (Figure 3), which indicates oxidative stress in the plants with changed TAA.

**Figure 6:**
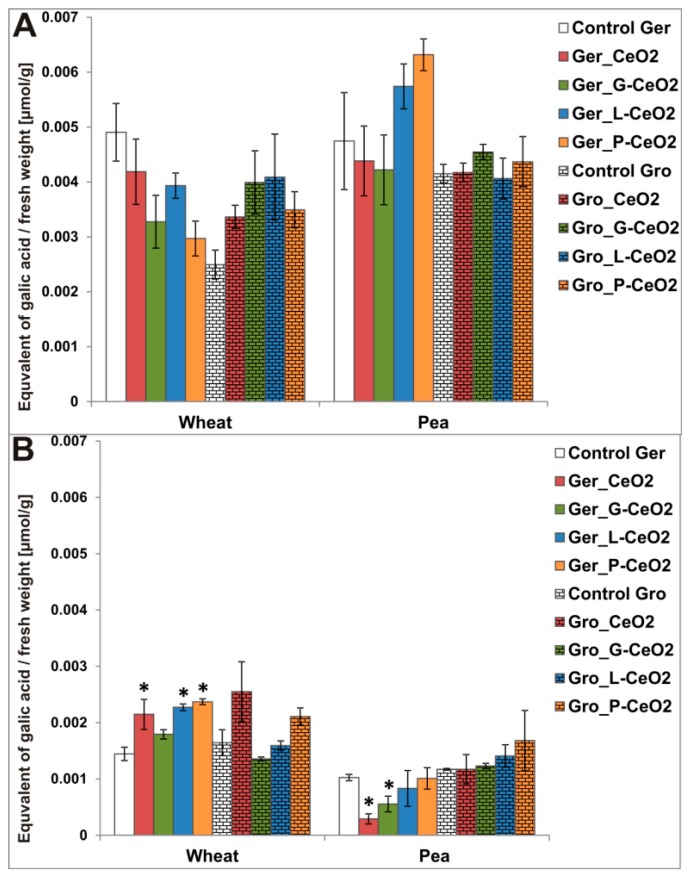
Total phenolic content (TPC) in shoots (**A**) and roots (**B**) of wheat and pea after Ger and Gro treatment with different nCeO_2_. Values are shown as mean ± SE; * indicates statistically significant differences in comparison with the corresponding control, *p* < 0.05.

The measurement of phenolic content, as an indicator of the plant defense capacity, is of great importance. According to the literary data, there are only a few reports about total phenolic content (TPC) in plants treated with nCeO_2_ (Supplementary Table S1). The effect of nCeO_2_ treatments on TPC in wheat and pea is presented in Figure 6. Similar to the effect on the shoot and root length, only the Ger treatment affected TPC, while the Gro treatment did not affect TPC in any of the tested plant species. In shoots, TPC was not significantly affected. In roots, TPC significantly increased in wheat after Ger_CeO_2_, Ger_L-CeO_2_, and Ger_P-CeO_2_, but decreased in pea after Ger_CeO_2_ and Ger_G-CeO_2_ treatments. The decrease of phenolic content in pea roots may indicate damage to the defense system due to its protecting role in reactive oxygen scavenging and metal chelation [44], while treatments with L-CeO_2_ and P-CeO_2_ alleviated this effect, rising the phenolics to the level in the control.

These results indicated that coated nCeO_2_ had a more significant impact on secondary metabolism, which can be related to a stronger defense reaction. Increased phenolics in wheat and decreased in pea can be related to different Ce concentration in these plant species. The high Ce concentration in wheat roots led to the increased phenolic content. Since there were no translocations from wheat roots to shoots for most of the studied nCeO_2_, phenolic content was not significantly increased in shoots. The increase in TPC in wheat after Ger treatment, as an indicator of defensive capacity, may open new possibilities in the use of nCeO_2_ as fertilizers/protective agents. In this context, the second generation should be screened.

**Figure 7:**
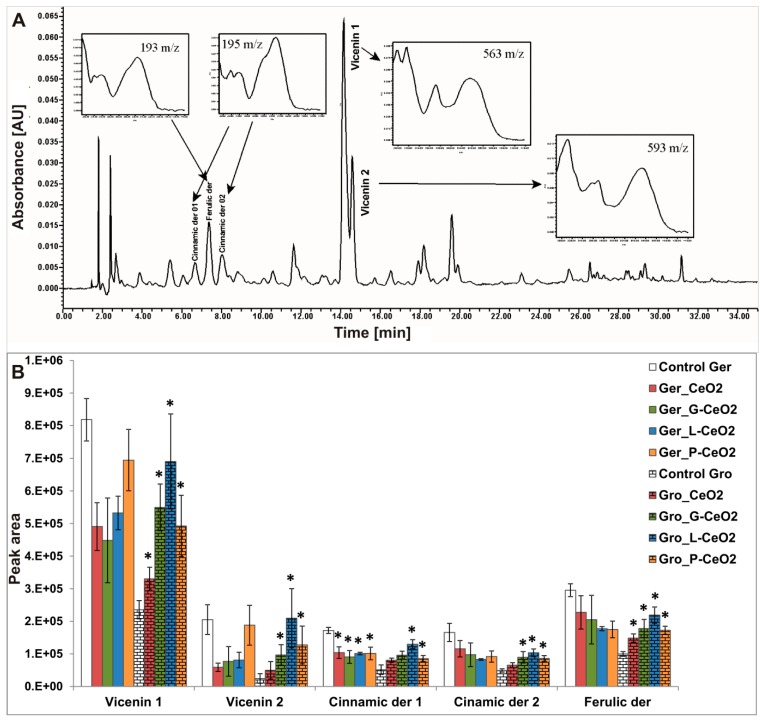
**(A**) High-performance liquid chromatography (HPLC) of the methanolic extracts of wheat shoots with ultraviolet–visible spectrometry (UV–Vis) of the dominant phenolic compounds; (**B**) Phenolic profile in wheat shoots after Ger and Gro treatment with different nCeO_2_. Values are shown as mean ± SE; * indicates statistically significant differences in comparison with the corresponding control, *p* < 0.05.

**Figure 8:**
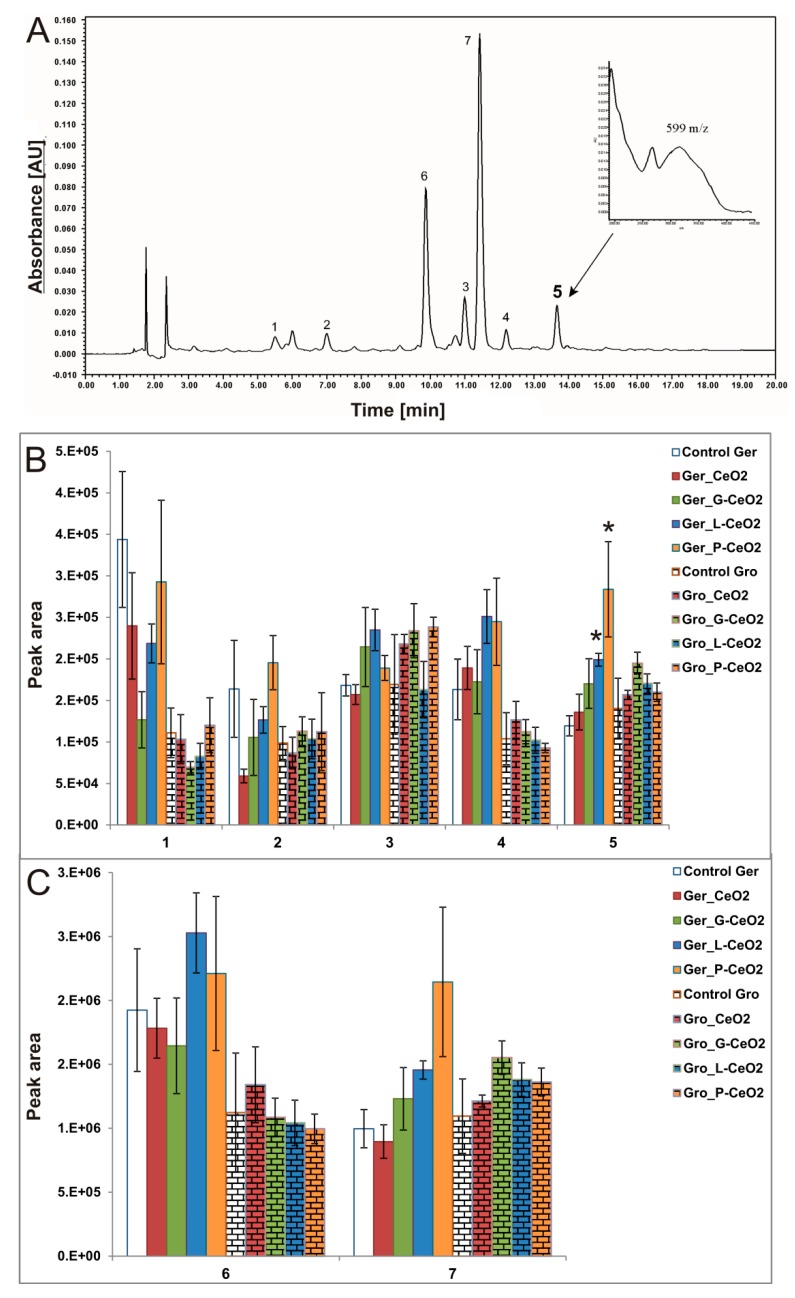
(**A**) HPLC chromatogram of methanolic extracts of pea shoots with UV–Vis spectra of phenolic compounds. (**B**) Unidentified compounds 1–5 and (**C**) unidentified compounds 6 and 7 detected in pea shoots after Ger treatment with different nCeO_2_. Values are shown as mean ± SE; * indicates statistically significant differences in comparison with the corresponding control, *p* < 0.05.

To our knowledge, no reported studies have analyzed phenolic profile in shoots of the nCeO_2_-treated plants. Phenolic patterns (Figure 7 and Figure 8) showed unchanged qualitative composition (type and number of phenolic compounds) in both tested plants, which indicates that the treatment with uncoated and coated nCeO_2_ did not significantly affect the plants’ phenolic profile.

In wheat, flavonoids vicenin 1 and 2 were identified as the dominant compounds, while two derivatives of hydroxycinnamic and one derivate of ferulic acid [45] were detected in minor amounts (Figure 7A). In pea, seven major unidentified compounds were found (Figure 8B). Comparing the peak areas for all treatments with the control in each plant, significant differences were observed in all identified phenolic compounds in wheat (Figure 7B) and in the unidentified compound 5 in pea (Figure 8B).

As for TAA, the Gro treatment more affected the phenolic profile than the Ger treatment in wheat shoots, while in pea shoots, only the Ger treatment was effective. In wheat shoots (Figure 7B), significant enhancement in peak areas was observed after the Gro treatment with all types of nCeO_2_ for vicenin 1 and a derivative of ferulic acid. The peak areas also increased after the treatment with all coated nCeO_2_ for vicenin 2 and derivative 2 of hydroxycinnamic acid, as well as after the treatment with Gro_L-CeO_2_ and Gro_P-CeO_2_ for derivative 1 of hydroxycinnamic acid. On the other hand, peak area reduction was observed only for derivative 1 of hydroxycinnamic acid after the Ger treatment with all types of nCeO_2_. The increased amount of all phenolic compounds in wheat shoots after Gro treatment may be a consequence of the high amount of accumulated Ce (Table 2) and was in agreement with the enhancement of TAA. The influence of coated nanoparticles was more expressed compared to the uncoated nanoparticles. Among the studied nanoparticles, L-CeO_2_ had the greatest influence on the phenolic profile. On the other hand, significant changes in pea were found only in compound 5 (Figure 8B) as a nondominant phenolic compound in pea shoot, after Ger_L-CeO_2_ and Ger_P-CeO_2_. These treatments did not affect TAA and TPC in pea. The observed changes in peak area in wheat and pea phenolic compounds (Figure 7 and Figure 8) may indicate that some specific phenolic compounds were induced by the treatments.

The possible effects of polysaccharides’ fragments as a signaling molecule in the case of coated nCeO_2_ should not be excluded. Plants may sense some polysaccharides as signals that stimulate their defense response. Also, the effects of pullulan coating may be partly due to its fragments, which are free of interaction with cerium. This was shown by ^1^H and ^13^C ssNMR (Supplementary Figure S1).

## 3. Materials and Methods

Ce(NO_3_)_3_∙6H_2_O, NaOH, glucose, 2,2′-azino-bis(3-ethylbenzothiazoline-6-sulphonic acid) (ABTS), H_2_O_2_, horseradish peroxidase type II (HRP) (150–250 units per mg solid), K_2_HPO_4_, KH_2_PO_4_, Na_2_CO_3_, gallic acid, 2N Folin-Ciocalteu reagent, CH_3_OH, and HNO_3_ were of analytical grade. All chemicals (including glucose) were obtained from Sigma Aldrich (St. Luis, USA).

As previously described, the polysaccharide levan was isolated from the *Bacillus licheniformis* NS032 strain (Genbank accession number JF826527) after growth on modified sucrose medium with ammonium–chloride as a nitrogen source [13,46]. The structure of the obtained levan was recently characterized in detail [47].

The polysaccharide pullulan was isolated after cultivation of the fungus *Aureobasidium pullulans* CH-1 strain. Its structure was investigated and confirmed as described previously [48].

### 3.1. Synthesis of the nCeO_2_ and Their Coated Homologues

Cerium-oxide nanoparticles were synthesized using the self-propagating room temperature (SPRT) method according to Milenković et al. (2018) [9]. In brief, starting materials (Ce(NO_3_)_3_∙6H_2_0 and NaOH) were hand-mixed in a mortar with a pestle for about 5–10 min. The obtained product was rinsed with deionized water three times and rinsed twice with ethanol in a centrifuge (Centurion 1020D, Chichester, UK) for 10 min at 4200 rpm. The powders were dried overnight at 70 °C. Cerium-oxide nanoparticles, synthesized by the SPRT method, were subsequently coated with three different carbohydrates (glucose/levan/pullulan) to obtain G-, L-, and P-CeO_2_, respectively. The mass ratio of carbohydrates to nCeO_2_ was 1:7, or 2.523 g of carbohydrates and 0.360 g of nCeO_2_. The obtained nanoparticles CeO_2_, G-, L- and P-CeO_2_ had an average hydrodynamic size of 385 nm, 235 nm, 216 nm, and 314 nm, and zeta potential values of 32.2, 31.3, 20.8, and 19.8, respectively [9]. The dry weights of the prepared nanocomposites were 2.379 g, 2.389 g, and 2.381g for G-, L-, and P-CeO_2_, respectively. A concentration of 200 mgL^−1^, as a concentration in the lower range of concentrations for hydroponics used in literature (10–10,000 mgL^−1^) [18], was used for the preparation of nanoparticle suspensions in all experiments. Before treatment, all nanoparticle suspensions were ultrasonicated in ultrasound bath Ultrasons HD (J. P. Selecta s.a., Barcelona, Spain) for 60 min at 120 W.

### 3.2. Characterization Methods

High-resolution transmission electron microscopy (HRTEM) and FEI Talos F200X were used for the characterization of nanoparticle suspensions. An X’Pert Pro MPD automated diffractometer (PANalytical, Almelo, The Netherlands) equipped with a Ge (111) primary monochromator (strictly monochromatic Cu-Kα radiation) and an X’Celerator detector (PANalytical, Almelo, The Netherlands) was used for the collection of powder diffraction patterns. For ssNMR spectroscopy, the samples were dried in an oven at 65 °C for 1 day. In the case of XPS analysis, the samples were directly deposited in the sample holder and dried in vacuum.

A Physical Electronic PHI 5700 spectrometer with a hemispherical multichannel detector was used for the XPS measurements by nonmonochromatic Mg-Kα radiation (300 W, 15 kV, and 1253.6 eV) to analyze the core-level signals of the elements of interest. The spectra were obtained with constant pass energy at 29.35 eV, using a 720-μm diameter circular analysis area. Analysis of the X-ray photoelectron spectra was performed by PHI ACESS ESCA-V6.0F software and processed by the MultiPak 8.2B package. The binding energy values were referenced to the adventitious carbon C 1*s* signal (284.8 eV). The binding energies were determined using the Shirley-type background and Gauss-Lorentz curves.

High-resolution ^13^C solid-state spectra for the different samples were recorded using the ramp ^1^H-^13^C CP-MAS sequence (cross-polarization and magic angle spinning) with proton decoupling during acquisition, respectively. The ^13^C cross-polarized magic angle spinning (CP-MAS) measurements were done at room temperature in a BrukerAvance-IIIHD 600 spectrometer equipped with a 3.2-mm MAS probe, and with the operating frequency for protons and carbons 600.09 MHz and 150.91 MHz, respectively. For the cross-polarization experiments in the ^13^C spectra, glycine was used as an external reference for the ^13^C spectra and for setting the Hartmann-Hahn matching condition. The contact time during CP was 2000 µs. Acquisition of the SPINAL64 sequence (small phase incremental alternation with 64 steps) was used for heteronuclear decoupling [49]. The spinning rate was 15 kHz for all the samples. The ^1^H-MAS experiments were performed in a 2.5-mm MAS probe with 600.09 MHz operating frequency for protons. ^1^H chemical shifts were indirectly referenced relative to neat tetramethyl silane, with powdered glycine as an external reference. The spinning rate was 30 kHz for all the samples.

### 3.3. Plant Growth and Treatments with Ce Nanoparticles

Commercially available seed material of monocotyledonous (wheat, from the local shop) and dicotyledonous plant (pea, from the local shop) were used. Sterilization of seeds was performed with 4% NaClO for 2 min and rinsed with distilled water (2–3 times for 1 min).

#### 3.3.1. Treatment During Seed Germination (Ger Treatment)

For each treatment (control, nCeO_2_ and G-, L-, and P-CeO_2_), four replicates of 20 seeds (of wheat and peas) were sown on and covered with filter paper moistened with 10 mL (200 mgL^−1^) treatment suspension in Petri dishes (20 Petri dishes per plant species). Germination percentage was measured daily using a radicle protrusion of more than 2 mm as a criterion [50]. Seed germination was performed in the dark at 25 °C for five days (wheat) and two days (pea).

After the treatment with different nCeO_2_ during germination, 32 seedlings per treatment of wheat/pea were transferred to the plastic vessels (6.5-cm height) containing nanoparticle-free Murashige&Skoog (MS) hydroponic medium [51]. The seedlings were grown in plant growth chamber LAE Electronic (Italy) for the next three weeks under a 16 h/8 h photoperiod at 25 °C. The light intensity was 150 µmolm^−2^s^−1^, measured by PAR Quantum Meter (UK). The plant height, root length, and fresh weight were measured. Roots were rinsed several times with Milli-Q water to eliminate adhered nanoparticles, as well as the components of the medium. After three weeks, the shoots and roots of eight plants (two plants per replicate) were collected, frozen in liquid nitrogen, and kept at −80 °C until the determination of TPC and TAA. The remaining 24 plants were used for the determination of elemental composition by ICP-OES analysis.

#### 3.3.2. Treatment during Plant Growth (Gro Treatment)

For this experiment, seed germination was performed in distilled water and 32 seedlings were transferred to the plastic vessels (6.5-cm height) containing 0.5 L MS/2 medium [51] in the presence of 200 mgL^−1^ nanoparticles (nCeO_2_, G-, L-, or P-CeO_2_) for three weeks. The nutrient solution was aerated by bubbling, which contributed to the maintenance of nCeO_2_ in solution, and was renewed weekly. The further procedure was the same as described in Ger treatment.

### 3.4. Sample Preparation for ICP-OES

Dry samples were digested with 96–98% HNO_3_ and 30% H_2_O_2_ (1:4) in Tecator digestion system and used for inductively coupled plasma-optical emission spectroscopy (ICP-OES) analysis [52]. After cooling to the room temperature, the digests were filtered using Whatman filter paper and volume was adjusted with Milli-Q water to 25 mL. Total Ce concentration was analyzed using ICP-OES Perkin Elmer Optima 4300 DV.

### 3.5. Extraction of Phenolics and Determination of TPC

To obtain extracts of phenolics, roots or shoots of eight plants (2 per sample, in 4 replicates) were homogenized in a mortar using liquid nitrogen. Homogenates were resuspended in 80% methanol in the 1:10 (m:v) ratio and stirred for 60 min at room temperature. Extracts were centrifuged for 5 min at 10,000 rpm and extracted phenolics were obtained in the supernatant.

Folin-Ciocalteu’s spectrophotometric procedure [53] was used for the determination of TPC in the samples. Folin-Ciocalteu reagent was mixed with phenolic extracts in 1 mL of total volume. Sodium carbonate solution was added after 3 min and the mixture was incubated at 25 °C for 60 min. The standard curve (0.1–2.0 mM) was constructed using gallic acid. Absorbance was read at 724 nm (2501 PC spectrophotometer, “Shimadzu”, Kyoto, Japan) and the results were presented as micromoles of gallic acid equivalents per gram of fresh weight.

### 3.6. Determination of TAA

TAA of the samples was measured based on the procedure of Cano et al. 1998 [54]. In brief, the reaction mixture contained 2 mM 2,2′-azino-bis(3-ethylbenzothiazoline-6-sulphonic acid (ABTS), 15 µM H_2_O_2_, 0.25 µM horseradish peroxidase (HRP) type II, and 20 µL of 80% methanol extract of the samples in 50 mM potassium–phosphate buffer, pH 7.5, in 1 mL of total volume. The assay was performed in four replicates per treatment at the temperature of 25 °C. The reaction was monitored at 730 nm (2501 PC spectrophotometer “Shimadzu”, Kyoto, Japan) until obtaining a stable absorbance of ABTS radical (ABTS˙^+^) formed in the reaction with HRP. After adding methanolic plant extracts, the decrease of absorbance due to ABTS˙^+^ depletion was used for calculation of TAA from the standard curve obtained with ascorbic acid (0.1–1 mM) as a universal antioxidant. The TAA was expressed as micromoles of ascorbic acid equivalents per gram of fresh weight.

### 3.7. Determination of Concentration of Individual Phenolics

Quantification of individual phenolic compounds was done by reversed phase HPLC analysis. Waters HPLC apparatus, consisting of 1525 binary pumps, a thermostat, 717+ autosampler connected to the Waters 2996 diode array, and EMD 1000 single quadrupole detector with ESI probe was used (Waters, Milford, MA, USA). The separation of phenolics was achieved on a Symmetry C-18 RP column (150 mm×4.6 mm packed with 5 µm diameter particles; Waters, Milford, MA, USA) and coupled to the appropriate guard column. Binary gradient elution, using A (0.1% formic acid) and B (acetonitrile), was performed at a flow rate of 1 mL/min, with slight modifications of gradient profile used to fine-tune separations for each analyzed species. The used gradient profiles were the variations of starting 10% B, followed by a linear rise up to 50% B in the next 30 min and 10 min inverse to 10% B, including an additional 5 min of equilibration time. The post-column flow splitter (ASI, Richmond, CA, USA) with a 5/1 split ratio was used to obtain reduced (0.2 mL/min) mobile phase inflow into the electrospray ionization (ESI) probe. For liquid chromatography/mass spectrometry (LC/MS) analysis, detection in negative scan mode (100–900 m/z) was used with following ESI source parameters: Capillary voltage at 3.0 kV; cone voltage at −35 V; extractor and RF lens voltages at 3.0 V and 0.2 V respectively. Source and desolvation temperatures were 130 and 400 °C, respectively, with N_2_ gas inflow of 500 L/h. Due to the lack of particular standards, quantification was performed using normalization of peak areas, obtained by division of the HPLC peak area for each compound with the peak area of the lowest detected amount. The data acquisition and spectral peak confirmation were carried out by the Waters Empower 2 Software (Waters, Milford, MA, USA).

### 3.8. Statistical Analysis

The raw data (Ce concentration, rate of germination and translocation in plants, shoot and root length, and TPC and TAA in shoot and root parts of wheat and pea treated with different nCeO_2_) were used as input variables. Exploratory and data analysis were performed by the IBM SPSS Statistics 20 software (IBM, New York, NY, USA). Subsequently, a one-way ANOVA test was applied to separately test the differences between root and shoot length in the two plant species, which were subjected to treatment with different nCeO_2_ (wheat and pea, n = 24). Intergroup comparisons (different treatments vs. control) were performed by the Bonferroni (for samples with equal variances) or Tamhane (for samples with unequal variances) post hoc test for two independent samples (*p* < 0.05). A non-parametric Kruskal-Wallis test for independent samples was used to test the differences in Ce concentration, rate of germination and translocation, TPC and TAA measured in root and shoot parts, and in phenolic profiles in shoots under the different treatments (n = 4 for both plant species). Post hoc intergroup comparisons of variables (between different treatments and control) for each plant separately were performed by the nonparametric Man–Whitney test at the level of the significance *p* < 0.05.

## 4. Conclusions

This was the first report on the effect of G-, L-, and P-CeO_2_ on the early stage of plant development and stress response, and on their impact on pea. Excluding G-CeO_2_, cerium concentration in both plant species was higher after the treatments with coated nanoparticles compared to the treatment with uncoated ones in all Ger treatments. Cerium translocation from roots to shoots was higher in pea than wheat. None of the treatments influenced the seed germination rate.

**Figure 9:**
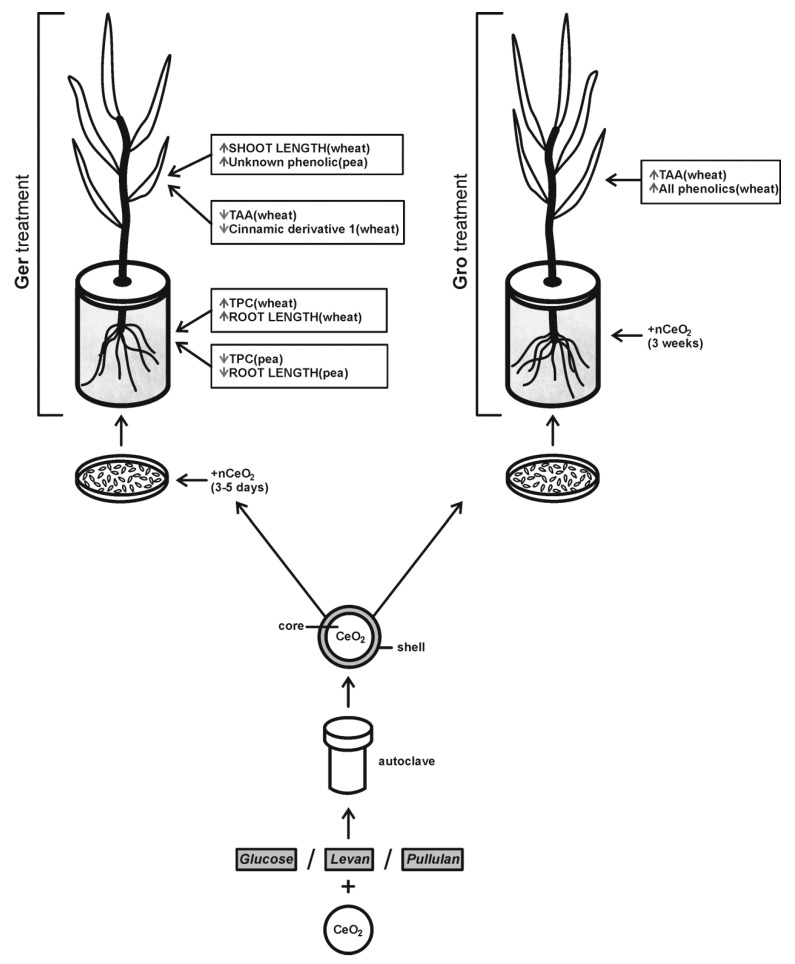
Scheme of the main effects of different nCeO_2_ on the studied plant species. Ger and Gro treatment: during germination and during growth, respectively. TPC and TAA: total phenolic content and total antioxidative activity, respectively. ↑, ↓ indicate an increase and decrease, respectively.

The main effects of the tested nCeO_2_ are summarized in the scheme on Figure 9. The exposure to all nCeO_2_ types had a higher influence on wheat, reflected in growth, TAA, TPC, and phenolic profile changes. In wheat roots, the TPC increased after Ger treatment, while growth and TAA were unaffected, meaning that TPC may boost the plant defense ability. This indicates that plant response to nCeO_2_ depends on the phase of development in which plants are exposed to them. The exposure of seeds to nanoparticles during germination affects the concentration of phenolic compounds and the elongation of shoots and roots, while exposure during growth induces an increase in non-enzymatic antioxidative response and modifies the profile of phenolic compounds.

We found that germination is a more sensitive phase of development to nCeO_2_ than growth. This may have an application in nano-priming technology for enhancing germination, seedling growth, and stress resistance. Among all used nanoparticles, L-CeO_2_ had the strongest effect on measured plants’ parameters.

## Figures and Tables

**Table 1:** Atomic compositions (at. %) of the Ce 3*d*, C 1*s*, and O 1*s* core levels, collected for the uncoated and coated nCeO_2_.

	Ce 3*d*	C 1*s*	O 1*s*
**CeO_2_**	19.97	21.48	58.55
**G-CeO_2_**	21.25	27.81	50.94
**L-CeO_2_**	19.45	29.31	51.24
**P-CeO_2_**	14.27	32.46	53.27

**Table 2:** Ce concentration (mg of Ce per kg of the dry mass of plant) in wheat and pea after germination (Ger) and three-week growth (Gro) treatments with different nCeO_2_ at 200 mgL^−1^.

	WHEAT		PEA	
	Shoot	Root	Shoot	Root
Control Ger	ND	ND	ND	ND
Ger_CeO_2_	0.05 ± 0.007	4.68 ± 0.051	ND	1.56 ± 0.076
Ger_G-CeO_2_	ND	3.16 ± 0.054 *	ND	1.33 ± 0.065
Ger_L-CeO_2_	ND	9.95 ± 0.058 *	ND	2.37 ± 0.076 *
Ger_P-CeO_2_	0.02 ± 0.006 *	10.67 ± 0.086 *	ND	3.61 ± 0.078 *
Control Gro	ND	ND	ND	ND
Gro_CeO_2_	51.36 ± 0.383	10892.86 ± 12.475	0.82 ± 0.044	88.42 ± 0.496
Gro_G-CeO_2_	26.60 ± 0.654 *	13675 ± 6.338 *	1.81 ± 0.066 *	147.16 ± 0.633 *
Gro_L-CeO_2_	33.13 ± 0.120 *	17522.73 ± 4.840 *	2.53 ± 0.056 *	117.22 ± 0.343 *
Gro_P-CeO_2_	25.47 ± 2.064 *	14593.75 ± 5.462 *	3.16 ± 0.03 *	88.46 ± 0.536

ND = not detected. “*” indicates values significantly different from the values for uncoated nCeO_2_. Detection limit was 0.05 mgL^−1^.

## Supplementary Materials

The following are available online at https://www.mdpi.com/2223-7747/8/11/478/s1, Figure S1: Left panel: ^13^C CP-MAS *ss*-NMR spectra (15 kHz) and middle panel: ^1^H-MAS *ss*-NMR spectra (30 kHz) for the levan (A), L-CeO_2_ (B), glucose (C), G-CeO_2_ (D), pullulan (E) and P-CeO_2_ (F) samples. Right panel: magnification of the ^1^H-MAS regions is shown in spectra B’, D’ and F’, Figure S2: Rate of translocation, expressed as a percentage, in wheat and pea after Ger treatment and Gro treatment, Figure S3: Rate of germination, expressed as a percentage, in wheat and pea, Table S1: Literature data about the effect of different coated and uncoated nCeO_2_ on various plant species, Table S2: ^13^C CP-MAS chemical shifts (ppm) for glucose, levan and pullulan materials.
