# Supplementary material for: Interaction of Carbohydrate Coated Cerium-Oxide Nanoparticles with Wheat and Pea: Stress Induction Potential and Effect on Development
Source: Plants (Basel). 2019 Nov 6;8(11):478. doi: 10.3390/plants8110478 (PMC6918407; doi:10.3390/plants8110478)
Supplement: Supplementary file 1 [file plants-08-00478-s001.pdf]

TABLE OF CONTENTS

Abstract……………………………………………………………………………………..1

1. INTRODUCTION………………………………………………………………………2

2. RESULTS AND DISCUSSION………………..……………………………………...2

2.1. nCeO$_2$ structural properties……………………..………………………………..2

2.2. Cerium Concentration and Translocation in Plants……………..…………………....8

2.3. Effect of nCeO$_2$ on Seed Germination and Plant Growth…………..…………………9

2.4. Antioxidative response to nCeO$_2$ treatments………………………..………………...10

3. MATERIALS AND METHODS……………………………………………………...17

3.1. Synthesis of the CeO$_2$ and their coated homologues……………………………………17

3.2. Characterization methods…………………………………………………………..…..18

3.3. Plant growth and treatments with Ce nanoparticles……………………..…………...18

3.3.1. Treatment during seed germination (Ger treatment)……………...…….………19

3.3.2. Treatment during plant growth (Gro treatment)…………………………....……..19

3.4. Sample preparation for ICP-OES……………………………………………………..19

3.5. Extraction of phenolics and determination of TPC…………………………….……..19

3.6. Determination of TAA..19

3.7. Determination of phenolic profile by HPLC……………………………….…………….20

3.8. Statistical analysis……………………….…………………………………………………20

4. CONCLUSIONS..21

Author Contributions Statement…………………………………………..…………..22

Acknowledgments……………………………………………………………..…..23

Conflicts of interest………………………………………………………..……..…23

REFERENCES…………………………………………………………………………24

Supplementary material

Interaction of carbohydrate coated cerium-oxide nanoparticles with wheat and pea: stress induction potential and effect on development

Ivana Milenković, Aleksandra Mitrović, Manuel Algarra, Juan M. Lázaro-Martínez, Enrique Rodríguez-Castellón, Vuk Maksimović, Slađana Z. Spasić, Vladimir P. Beškoski, Ksenija Radotić

1. INTRODUCTION

Supplementary Table S1. Literature data about the effect of different coated and uncoated $nCeO_2$ on various plant species

Nanoparticle	Species	Dose	Treatment duration	Effects	References
Citric acid-CeO_2	*Solanum lycopersicum*	62.5 and 500 mg kg^{-1}	210 days	Decrease dry weight, total sugars, starch and reducing sugars	[1]
		125 mg kg^{-1}		Decrease starch and reducing sugars	
		–250 mg kg^{-1}		Reduce dry weight and total sugars	
	Raphanus sativus	200 mg L^{-1}	5 days	Increase root biomass	[2]
CeO_2	*Medicago arborea*				[3]
		100 - 400 mg L^{-1}	4 weeks	Increase root length	
		200 mg L^{-1}		Decrease root dry weight	

CeO_2	*Lactuca sativa*	250 - 1000 μg mL^{-1}	Few days	Decrease root length	[4]
	Lolium Perenne	500 - 1000 μg mL^{-1}		Increase root length	
	Solanum lycopersicum	500 μg mL^{-1}		Decrease root length	
CeO_2	*Cucumis sativus*	2000 mg L^{-1}	9 days	Decrease seed germination	[5]
		500 - 4000 mg L^{-1}		Increase root growth	
		500 - 4000 mg L^{-1}		Increase shoot elongation	
	Solanum lycopersicum	2000 mg L^{-1}	6 days	Decrease seed germination	
		1000 and 4000 mg L^{-1}		Decrease root growth	
	Zey mays	500 - 2000 mg L^{-1}	8 days	Decrease seed germination	
		4000 mg L^{-1}		Increase root growth	
		2000 and 4000 mg L^{-1}		Decrease shoot elongation	

	Medicago sativa	2000 and 4000 mg L^{-1}	9 days	Decrease root growth	
		500 and 1000 mg L^{-1}		Increase shoot elongation	
CeO_2	*Glycine max*	500 - 4000 mg L^{-1}	5 days	Decrease rooth growth	[6]
CeO_2	*Oryza sativa*	62.5 - 500 mg L^{-1}	10 days	Change enzyme activity in shoots and roots	[7]
CeO_2	*Oryza sativa* grains	500 mg kg^{-1}	135 days	Decrease antioxidant activity and TPC	[8]
CeO_2	*Raphanus sativus*	500 mg kg^{-1}	12 days	Decrease seed germination	[9]
		125 mg kg^{-1}	40 days	Increase TAA in leaves	
CeO_2	*Cucumis sativus*	800 mg kg^{-1}	53 days	Decrease TPC	[10]

2. RESULTS AND DISSCUSSION

In the present work, the Ce ions induced a strong relaxation for the different ^{13}C nuclei present in the starting materials avoiding the detection of resonance signals, even when not all the carbons were interacting or in the surrounding of the $nCeO_2$. In this sense, the Ce ions were well dispersed in the entire materials, taking into account that practically all the carbon signals were vanished due to the interaction or proximity with Ce. In the case of P-CeO_2, the one remained ^{13}C resonance signal was still present at 73.4 ppm, which was the most intense signal in the ^{13}C CP-MAS spectrum of the pullulan sample (Figure. 4 upper panel). In the rest of the sample containing Ce ions, the use of ^{13}C direct polarization or ^{13}C CP-MAS using short contact times (50-100 μs) strategies gave rise to the same results.

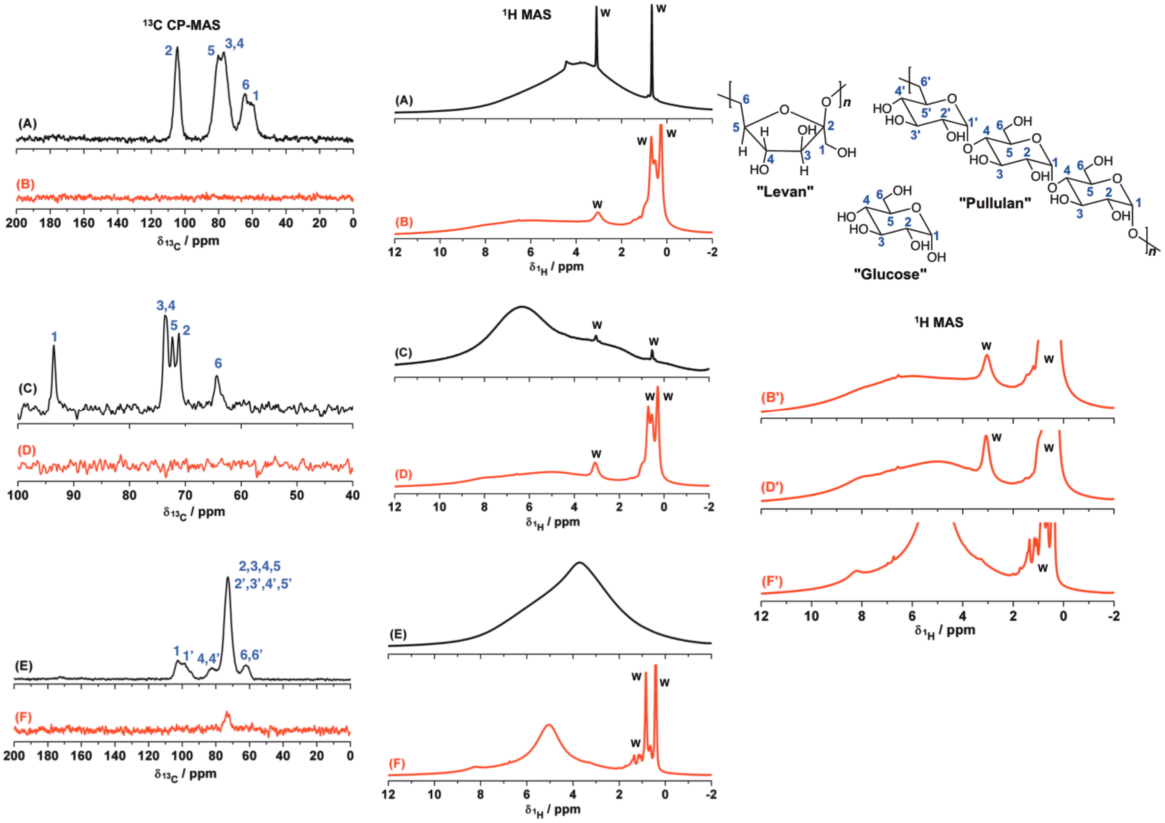

Supplementary Figure S1. Left panel: ^{13}C CP-MAS *ss*-NMR spectra (15 kHz) and middle panel: ^{1}H-MAS *ss*-NMR spectra (30 kHz) for the levan (A), L-CeO_2 (B), glucose (C), G-CeO_2 (D), pullulan (E) and P-CeO_2 (F) samples. Right panel: magnification of the ^{1}H-MAS regions is shown in spectra B', D' and F'.

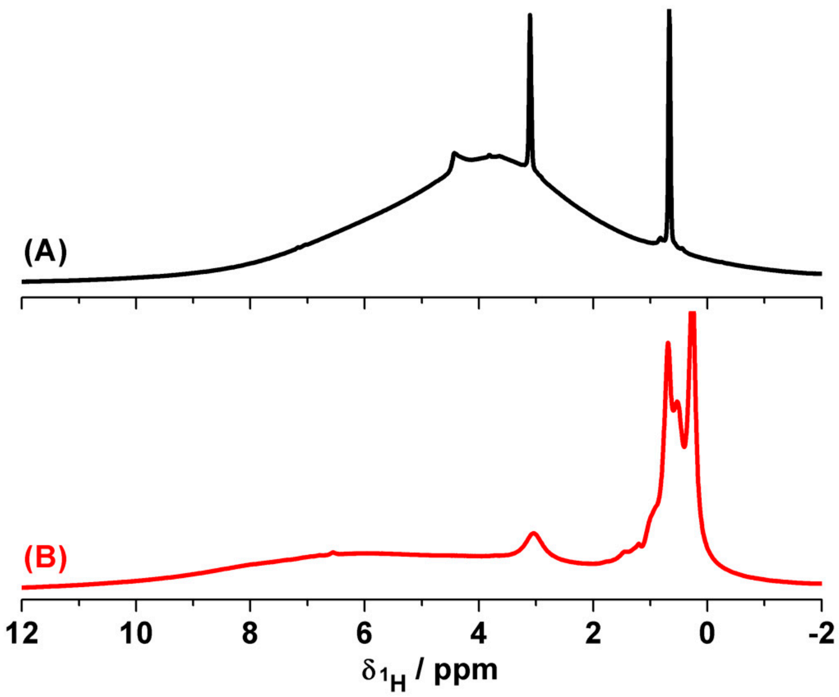
(A)
(B)
12
10
8
6
4
2
0
-2
$\delta_{^1H}$ / ppm

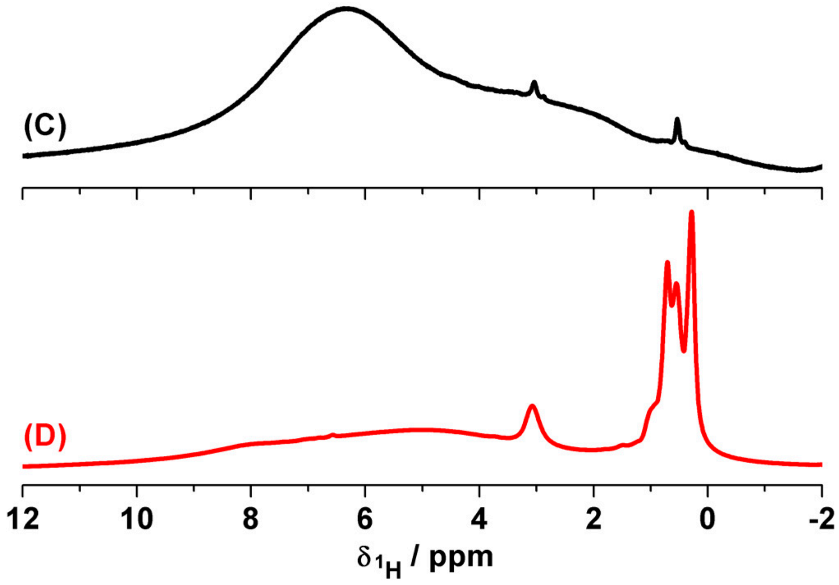
(C)
(D)
12
10
8
6
4
2
0
-2
$\delta_{^1H}$ / ppm

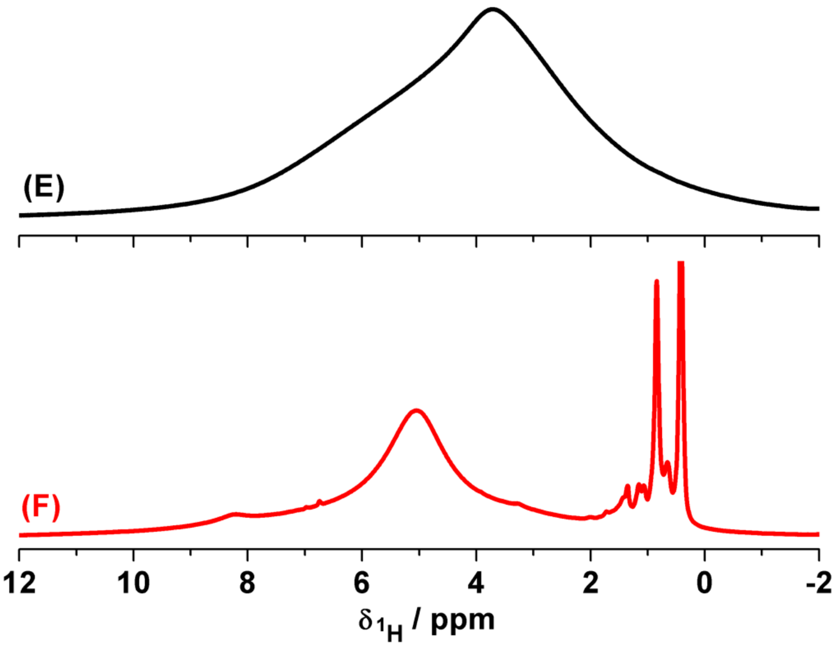

Supplementary Figure S1 A, B, C, D, E and F. ^{1}H-MAS *ss*-NMR spectra (30 kHz) for the levan (A), L-CeO_2 (B), glucose (C), G-CeO_2 (D), pullulan (E) and P-CeO_2 (F)

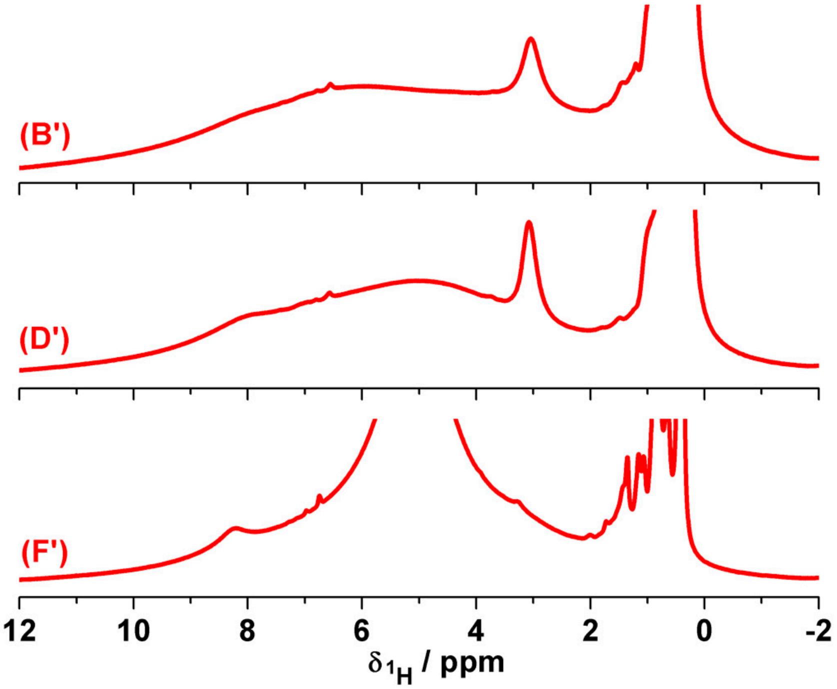

Supplementary Figure S1 B′, D′ and F′. Magnification of the ^{1}H-MAS regions is shown in spectra B′ (L-CeO_2), D′ (CeO_2) and F′ (P-CeO_2).

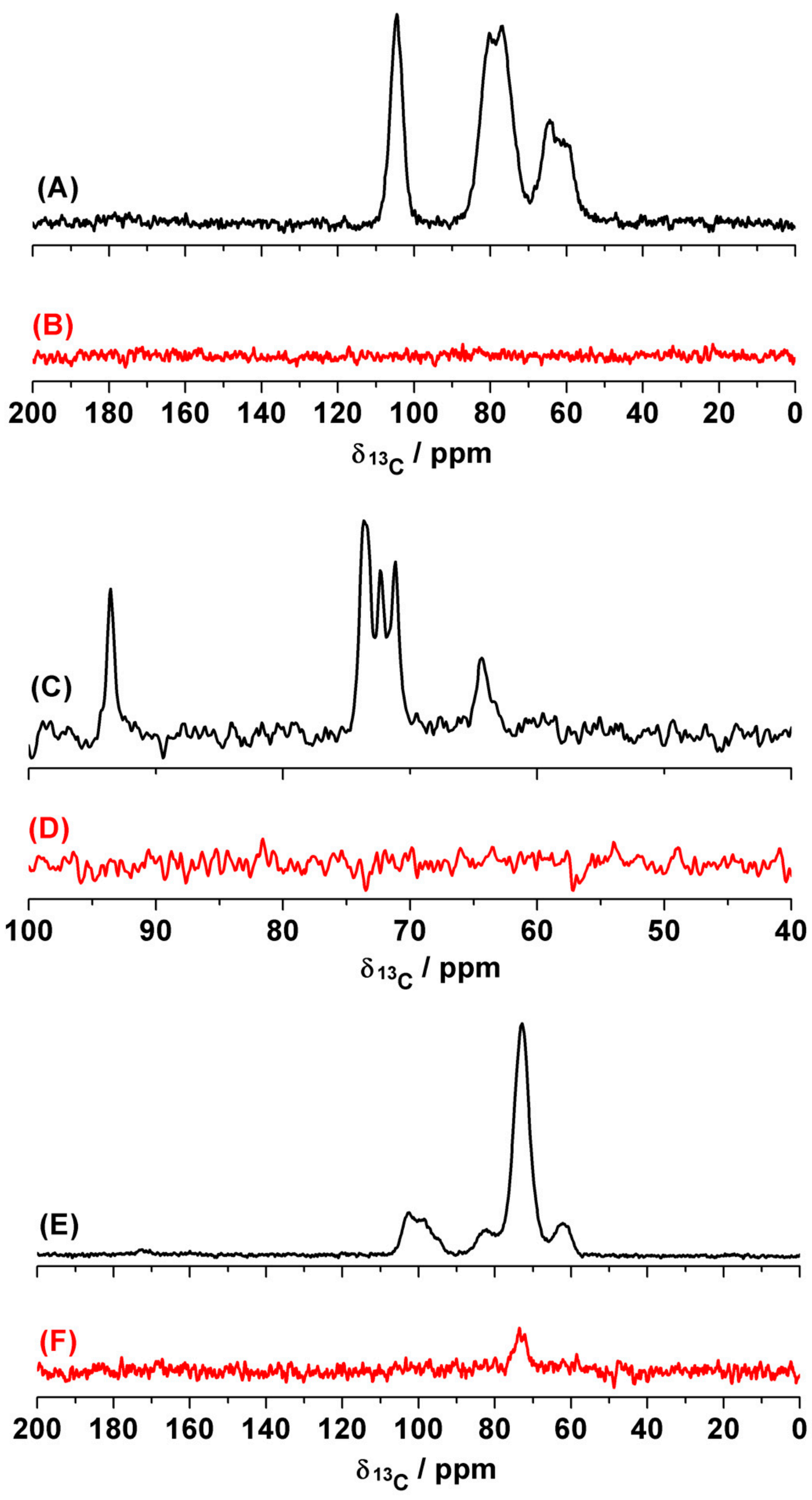
(A)
(B)
200 180 160 140 120 100 80 60 40 20 0
$\delta_{^{13}C}$ / ppm
(C)
(D)
100 90 80 70 60 50 40
$\delta_{^{13}C}$ / ppm
(E)
(F)
200 180 160 140 120 100 80 60 40 20 0
$\delta_{^{13}C}$ / ppm

Supplementary Figure S1 A, B, C, D, E and F. ^{13}C CP-MAS *ss*-NMR spectra (15 kHz) for the levan (A), L-CeO_2 (B), glucose (C), G-CeO_2 (D), pullulan (E) and P-CeO_2 (F) samples.

The assignment of the ^{13}C NMR signals was done in comparison with previous results in the *ss*-NMR for glucose and in the solution-state NMR for levan and pullulan materials. To the best of our knowledge, this is the first work which represents *ss*-NMR for levan and pullulan compounds. Related to pullulan material, the anomeric carbons ($C_{1\text{-}1'}$) were differentiated from the rest of the hydrocarbon chain corresponding to the different environments of $\alpha_{1\rightarrow4}$ (C_1) and $\alpha_{1\rightarrow6}$ ($C_{1'}$) bounds where these carbons were involved (Table 2).

Supplementary Table S2. ^{13}C CP-MAS chemical shifts (ppm) for glucose, levan and pullulan materials.

**CarbonAtom**[a]	**Glucose**	**Levan**	**CarbonAtom**[a]	**Pullulan**
**C_1**	93.5	60.7	C_1 / $C_{1'}$	102.5 / 98.3
**C_2**	71.1	104.6	C_2 / $C_{2'}$	73.0
**C_3**	73.5	76.8	C_3 / $C_{3'}$	73.0
**C_4**	73.5	76.8	C_4 / $C_{4'}$	82.5
**C_5**	72.2	80.6	C_5 / $C_{5'}$	73.0
**C_6**	64.3	64.6	C_6 / $C_{6'}$	62.1

With the aim to get more structural information related to the nanocomposites with Ce ions, ^1H-MAS *ss*-NMR experiments were done and the results are shown in Figure. 4. The ^1H-MAS spectrum for the P-CeO_2 shows two ^1H resonance signals at 5.1 and 8.2 ppm ascribed to -C**H**-OH and –O**H**hydrogens, respectively. However, the rest of the ^1H signals in G-CeO_2 and L-CeO_2 were highly affected and vanished by the paramagnetic effect of Ce ions with the exception of the water populations. These results indicated that in P-CeO_2 sample there are some biopolymer regions that did not interact with the nCeO_2, explaining that a ^{13}C signal at 73.4 ppm among the ^1H signals at 5.1 and 8.2 ppm are still present in the NMR spectra. Even when the linewidth of the ^1H-MAS spectra was broad and contained some water signals indicated as 'W' in the starting materials without Ce ions, the interaction with Cevanished the main proton signal in each sample (Figure. 4, middle and bottom panel). Interestingly, the ^1H-MAS spectra of any of the nCeO_2 present similar patterns of water structuration at a proton chemical shift (δ^1H) of 0-2 ppm. These water populations were related to weakly interactions among water molecules. In the starting materials without Ce ions, some water clusters are present at a δ^1H = 1 and 3 ppm [11]. However, weakly associated water (WAW) molecules interact with the nanostructure surfacesthrough the hydrogen bonds or electrostatically. The *nano*-structuration of the different materials with Ce ions involved also the organization of water molecules with a new, similar and well-resolved pattern of ^1H signals in the samples with CeO_2. In this sense, the nCeO_2 particles were highly homogenous dispersed in the G-CeO_2 and L-CeO_2 samples due to the vanishing of all ^1H and ^{13}C-NMR signals considering that the *ss*-NMR experiments were done in the same conditions.

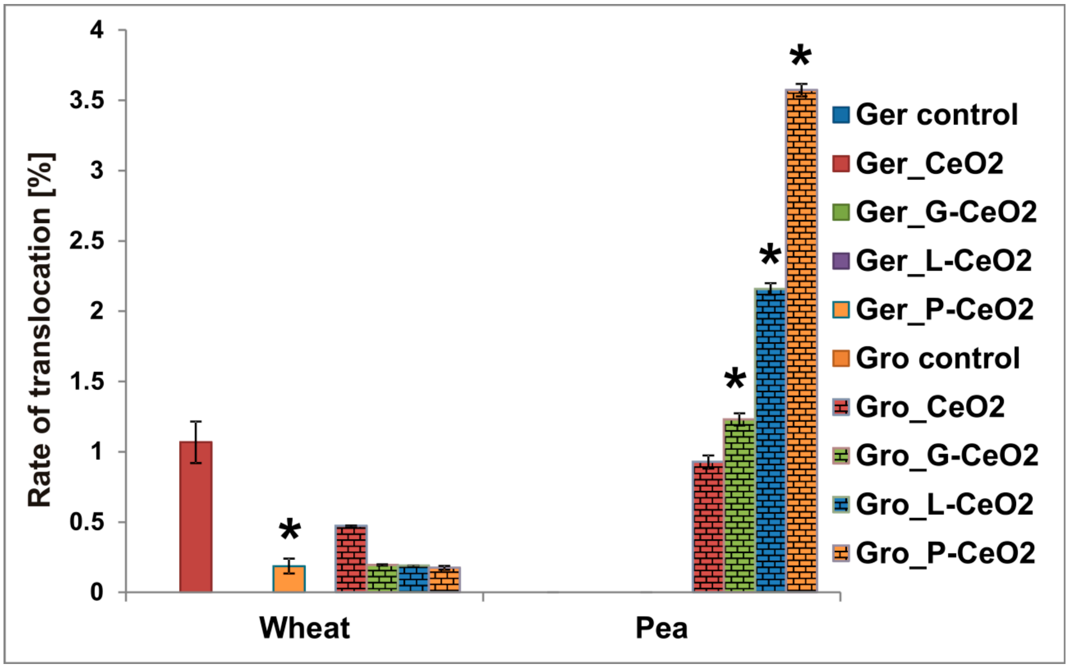

Supplementary Figure S2. Rate of translocation, expressed as a percentage, in wheat and pea after Ger treatment and Gro treatment. Values are shown as mean ± SE; * indicates a statistically significant difference in comparison with the corresponding control, $p<0.05$

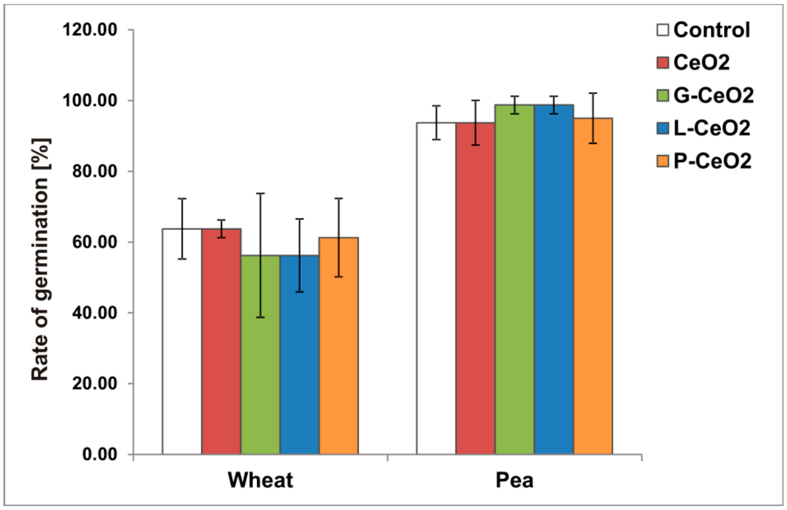

Supplementary Figure S3. Rate of germination, expressed as a percentage, in wheat and pea. Values are shown as mean ± SE; * indicates a statistically significant difference in comparison with the corresponding control, $p<0.05$

REFERENCES

1. Barrios, A.C.; Medina-Velo, I.A.; Zuverza-Mena, N.; Dominguez, O.E.; Peralta-Videa, J.R.; Gardea-Torresdey, J.L. Nutritional quality assessment of tomato fruits after exposure to uncoated and citric acid coated cerium oxide nanoparticles, bulk cerium oxide, cerium acetate and citric acid. *Plant Physiol. Biochem.* **2017**, *110*, 100-107.
2. Trujillo-Reyes, J.; Vilchis-Nestor, A.; Majumdar, S.; Peralta-Videa, J.; Gardea-Torresdey, J. Citric acid modifies surface properties of commercial CeO2 nanoparticles reducing their toxicity and cerium uptake in radish (Raphanus sativus) seedlings. *J. Hazard. Mater.* **2013**, *263*, 677-684.
3. Gomez-Garay, A.; Pintos, B.; Manzanera, J.A.; Lobo, C.; Villalobos, N.; Martín, L. Uptake of CeO 2 nanoparticles and its effect on growth of Medicago arborea in vitro plantlets. *Biol. Trace Elem. Res.* **2014**, *161*, 143-150.
4. Andersen, C.P.; King, G.; Plocher, M.; Storm, M.; Pokhrel, L.R.; Johnson, M.G.; Rygiewicz, P.T. Germination and early plant development of ten plant species exposed to titanium dioxide and cerium oxide nanoparticles. *Environ. Toxicol. Chem.* **2016**, *35*, 2223-2229.
5. López-Moreno, M.L.; de la Rosa, G.; Hernández-Viezcas, J.A.; Peralta-Videa, J.R.; Gardea-Torresdey, J.L. XAS corroboration of the uptake and storage of CeO2 nanoparticles and assessment of their differential toxicity in four edible plant species. *J. Agric. Food Chem.* **2010**, *58*, 3689.
6. López-Moreno, M.L.; de la Rosa, G.; Hernández-Viezcas, J.Á.; Castillo-Michel, H.; Botez, C.E.; Peralta-Videa, J.R.; Gardea-Torresdey, J.L. Evidence of the differential biotransformation and genotoxicity of ZnO and CeO2 nanoparticles on soybean (Glycine max) plants. *Environ. Sci. Technol.* **2010**, *44*, 7315-7320.
7. Rico, C.M.; Hong, J.; Morales, M.I.; Zhao, L.; Barrios, A.C.; Zhang, J.-Y.; Peralta-Videa, J.R.; Gardea-Torresdey, J.L. Effect of cerium oxide nanoparticles on rice: a study involving the antioxidant defense system and in vivo fluorescence imaging. *Environ. Sci. Technol.* **2013**, *47*, 5635-5642.
8. Rico, C.M.; Morales, M.I.; Barrios, A.C.; McCreary, R.; Hong, J.; Lee, W.-Y.; Nunez, J.; Peralta-Videa, J.R.; Gardea-Torresdey, J.L. Effect of cerium oxide nanoparticles on the quality of rice (Oryza sativa L.) grains. *J. Agric. Food Chem.* **2013**, *61*, 11278-11285.
9. Corral-Diaz, B.; Peralta-Videa, J.R.; Alvarez-Parrilla, E.; Rodrigo-García, J.; Morales, M.I.; Osuna-Avila, P.; Niu, G.; Hernandez-Viezcas, J.A.; Gardea-Torresdey, J.L. Cerium oxide nanoparticles alter the antioxidant capacity but do not impact tuber ionome in Raphanus sativus (L). *Plant Physiol. Biochem.* **2014**, *84*, 277-285.
10. Zhao, L.; Peralta-Videa, J.R.; Rico, C.M.; Hernandez-Viezcas, J.A.; Sun, Y.; Niu, G.; Servin, A.; Nunez, J.E.; Duarte-Gardea, M.; Gardea-Torresdey, J.L. CeO2 and ZnO nanoparticles change the nutritional qualities of cucumber (Cucumis sativus). *J. Agric. Food Chem.* **2014**, *62*, 2752-2759.
11. Crespi, A.F.; Byrne, A.J.; Vega, D.; Chattah, A.K.; Monti, G.A.; Lázaro-Martínez, J.M.

    Generation and stability of the gem-diol forms in imidazole derivatives containing

carbonyl groups. Solid-state NMR and single-crystal X-ray diffraction studies. *J. Phys. Chem. A* **2018**, *122*, 601-609.
